# Development and validation of a nomogram for predicting tracheostomy risk in traumatic cervical spinal cord injury

**DOI:** 10.3389/fneur.2025.1684974

**Published:** 2026-01-15

**Authors:** Weiting Chen, Xiaoshuang Jiang, Xixi Guo, Jiuzhou Lin, Min Tang, Nanlin Dou

**Affiliations:** 1 Department of Emergency Medicine, The First People’s Hospital of Linhai, Taizhou, Zhejiang, China; 2 Department of Emergency and Intensive Care Unit, Linhai Branch of Second Affiliated Hospital, Zhejiang University School of Medicine, Taizhou, China; 3 Department of Traditional Chinese Medicine, The First People’s Hospital of Linhai, Lishui, Zhejiang, China; 4 Department of Intensive Care Medicine, Lishui Traditional Chinese Medicine Hospital Affiliated to Zhejiang Chinese Medical University, Lishui, Zhejiang, China

**Keywords:** spinal cord injury, cervical vertebrae, airway management, logistic models, intensive care units

## Abstract

**Background:**

Tracheostomy is common in traumatic cervical spinal cord injury (TCSCI) because of respiratory complications, yet objective tools to estimate individual risk remain limited.

**Methods:**

In this single-center retrospective cohort at the Second Affiliated Hospital, Zhejiang University School of Medicine, we enrolled 308 consecutive ICU admissions with TCSCI (January 2018–March 2023) and randomly split the cohort 7:3 (outcome-stratified) into training (*n* = 215) and validation (*n* = 93) sets. Candidate admission predictors were screened with Least Absolute Shrinkage and Selection Operator and then entered into multivariable logistic regression to construct a nomogram. Model performance included discrimination (AUC with bootstrap 95% CIs, 2,000 resamples), calibration (intercept, slope, Brier), and decision curve analysis (DCA). A prespecified clinical threshold of 0.30 was used to summarize sensitivity and specificity.

**Results:**

Five independent predictors were retained—smoking history, thoracic injury, BMI ≥ 25 kg/m^2^, cervical dislocation, and ASIA grade (A vs. B-D). The model showed strong discrimination (AUC 0.844, 95% CI 0.788–0.896 in training; 0.903, 95% CI 0.823–0.966 in validation) and good calibration. At the 0.30 threshold, performance was Sensitivity 0.781/Specificity 0.725 (training) and Sensitivity 0.812/Specificity 0.852 (validation); DCA demonstrated greater net benefit than “treat all/none” across threshold 0.10–0.70.

**Conclusion:**

A parsimonious, five-factor nomogram based on routine admission data provides accurate, clinically interpretable stratification of tracheostomy risk in TCSCI. Clear reporting of ASIA coding and a prespecified decision threshold enhance bedside usability. Prospective, multi-center external validation is warranted.

## Background

Traumatic cervical spinal cord injury (TCSCI) is a devastating condition associated with substantial morbidity, long-term disability, and mortality (1). Reported evidence indicates that its incidence has risen in recent decades—largely due to motor vehicle collisions, falls, and sports injuries—which has placed increasing demands on healthcare systems (2). Patients with TCSCI frequently develop severe neurological deficits and respiratory complications, often requiring intensive care unit (ICU) admission and advanced ventilatory support (3).

Against this clinical backdrop, the timing of tracheostomy in patients with TCSCI is challenging in clinical decisions. Tracheostomy is considered for prolonged mechanical ventilation, airway protection, and secretion clearance (4). In selected populations, early tracheostomy may shorten the duration of ventilation and ICU stay and reduce ventilator-associated pneumonia. However, optimal timing and criteria in TCSCI remain debated, and both delayed and unnecessary procedures carry added risks and costs (5–9). Current decisions rely largely on clinician judgment integrating neurological status, injury level, and comorbidities (10). Although prior studies using multivariable logistic regression and classification and regression trees (CART) have identified risk factors, a widely accepted, validated prediction tool specific to TCSCI is lacking (11–13).

There is currently no concise, externally interpretable tool that converts routinely available admission data into an individualized risk estimate to guide early care pathways in TCSCI. Providing a calibrated bedside probability shortly after ICU admission could (i) prompt timely tracheostomy or airway consultation, (ii) inform resource allocation and workflow planning (e.g., anticipated ventilator days, operating room or bronchoscopy scheduling), and (iii) support family counseling and multidisciplinary coordination. Nomograms translate regression results into individualized risk estimates and are increasingly used at the bedside for their transparency and practicality (14–16). Accordingly, we aimed to develop and internally validate a nomogram based on routinely available admission variables to estimate an individual patients’s likelihood of tracheostomy in TCSCI.

## Materials and methods

### Study design and setting

We conducted a single-center, retrospective cohort study in the ICU of the Second Affiliated Hospital, Zhejiang University School of Medicine. Potential cases were identified using the International Classification of Diseases, Tenth Revision (ICD-10) codes for traumatic spinal cord injury with cervical involvement and were subsequently verified against discharge summaries to ensure case ascertainment. The study period was January 1, 2018–March 31, 2023. Ethical approval was granted by the institutional review board (Approval No. 2023-0287). This design provided a consistent pathway for exposure, covariate, and outcome capture across all admissions.

### Outcome and clinical decision-making

**Figure 1:**
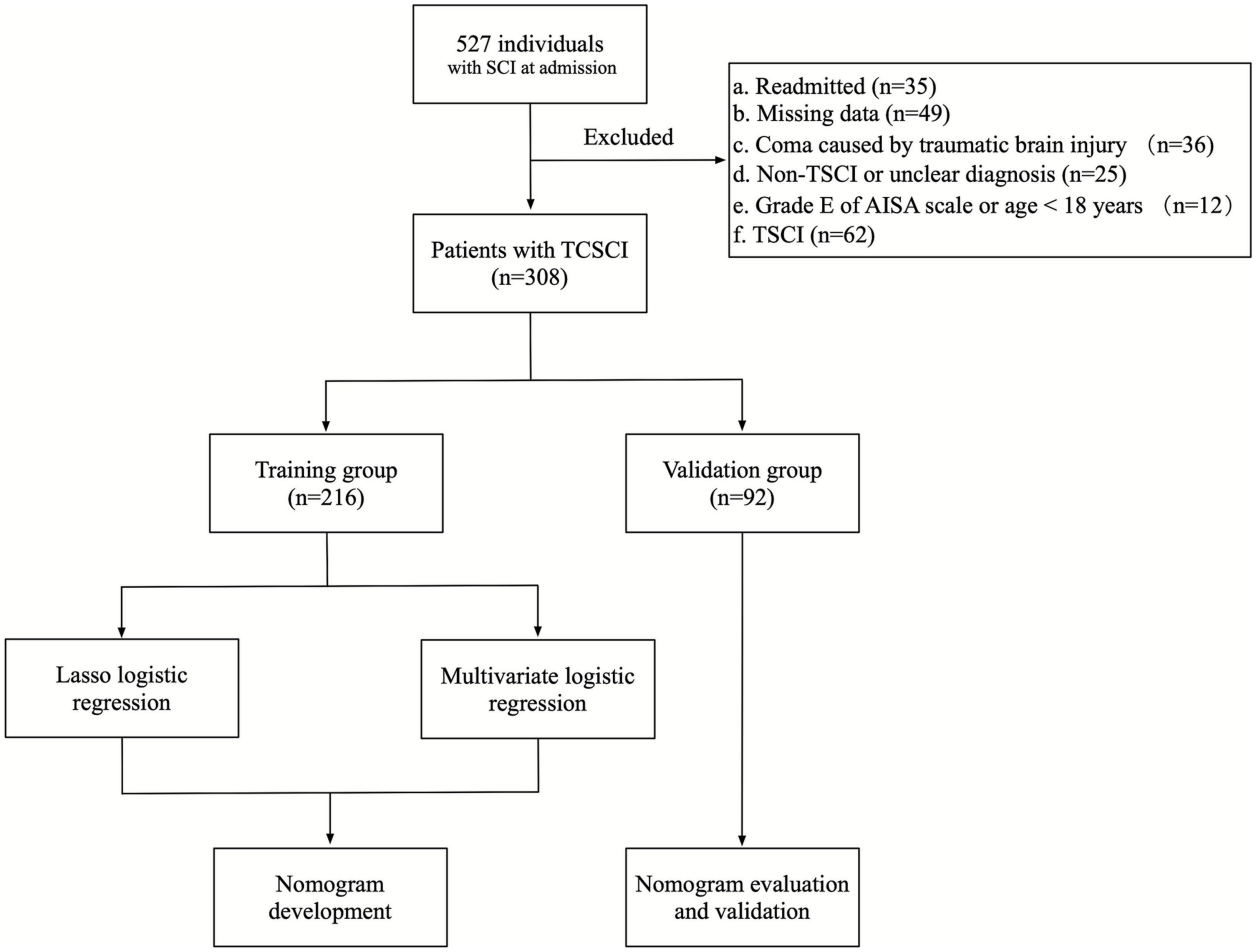
Study flow diagram.

The primary outcome was tracheostomy performed during the index admission. Decisions were made jointly by a spine surgeon and an ICU physician in the acute phase, based on comprehensive assessment of neurological and respiratory status, age, concomitant injuries, and other relevant indicators. Tracheostomy was considered when prolonged mechanical ventilation was anticipated (>14 days), or when airway protection or refractory secretion management was required. All evaluations and decisions were performed by senior attending physicians to promote consistency. The study workflow and patient selection are shown in Figure 1.

### Study participants

Electronic medical records of ICU patients with TCSCI during the study period were screened by three independent reviewers using a predefined abstraction form. Any discrepancies were adjudicated by consensus to ensure data integrity.

#### Inclusion criteria

Adults (≥18 years) presenting to our hospital and admitted to the ICU at initial evaluation were eligible. Diagnosis of TCSCI required all of the following: (i) a compatible traumatic mechanism (e.g., motor vehicle collision, fall, sports-related trauma); (ii) neurological deficits consistent with a cervical lesion on admission according to the International Standards for Neurological Classification of Spinal Cord Injury (ISNCSCI), with American Spinal Injury Association (ASIA) Impairment Scale grading recorded; and (iii) radiological confirmation on MRI and/or CT demonstrating acute cervical spinal injury (e.g., fracture/dislocation, cord edema/contusion/ hemorrhage, compressive lesions, or ligamentous instability) corresponding to the clinical level. Cases were first identified using ICD-10 codes and then verified by senior clinicians at admission.

#### Exclusion criteria

Patients were excluded for: readmission to the ICU during the study period (index admission only); non-traumatic etiologies (e.g., degenerative, neoplastic, infectious, or vascular) involving the spinal cord; age <18 years; intervertebral disc disorders without trauma; spinal fractures without cord involvement; pre-existing neuromuscular disorders; concomitant craniocerebral trauma that precluded reliable neurological assessment; tracheostomy performed prior to transfer or at another institution; missing or incomplete key clinical data; ASIA grade E; or death prior to hospital admission.

### Clinical management and ICU referral

Patients were referred to the ICU based on prespecified clinical triggers for respiratory monitoring and ventilatory support, including high cervical injury (C1–C4) with ASIA grade A–B; progressive respiratory distress or hypoxemia; worsening pulmonary mechanics (e.g., vital capacity <15–20 mL/kg or negative inspiratory force less negative than −20 cmH₂O); ineffective cough with copious secretions or recurrent atelectasis; aspiration risk or pneumonia; or the need for close hemodynamic or neurologic monitoring after multisystem trauma or surgery. Initial management followed a stepwise approach, including oxygen therapy, airway toileting, trial of noninvasive ventilation when appropriate, and escalation to invasive mechanical ventilation if noninvasive support failed or airway protection was required, according to clinician judgment.

### Data collection and variable definitions

We abstracted demographic and clinical variables at ICU admission, including age, sex, smoking history, cervical dislocation, diabetes mellitus, hypertension, pre-existing pulmonary disease, treatment strategy (operation, mannitol, corticosteroids), ASIA Impairment Scale grade, neurological level of injury (NLI), Injury Severity Score (ISS), body mass index (BMI), and concomitant injuries.

ASIA grades were assigned according to the ISNCSCI. For modeling, grades were coded as A = 0 and B–D = 1, and results are reported as ASIA A versus B–D for clinical interpretability (17). NLI was dichotomized as C1–C4 (high cervical) versus C5–C8 (low cervical). BMI was categorized as <25 vs. ≥ 25 kg/m^2^ (18). ISS was dichotomized as ≥16 vs. < 16 (19).

Cervical dislocation was defined as traumatic facet-joint displacement confirmed by CT and/or MRI. Pre-existing pulmonary disease included chronic obstructive pulmonary disease, bronchial asthma, and restrictive ventilatory disorders. Smoking history followed World Health Organization criteria (continuous or cumulative tobacco use for ≥6 months over the lifetime) (20).

Concomitant injuries comprised traumatic brain injury, maxillofacial fractures, thoracic trauma, abdominal trauma, pelvic fractures, and limb fractures or dislocations.

All modeling variables were routine admission items, and records with incomplete or unclear information were excluded during screening; consequently, the analytic cohort had 0% missingness for modeling variables and the outcome.

### Statistical analysis

Patients were randomly assigned to training and validation cohorts in a 7:3 stratified split by outcome (prespecified seed). Descriptive statistics summarized baseline characteristics. Group comparisons in the training cohort were exploratory (χ^2^ test or Fisher’s exact test, as appropriate) and not used for model selection. Candidate predictors available at ICU admission were preselected using the Least Absolute Shrinkage and Selection Operator (LASSO) and then entered a multivariable logistic regression with stepwise reduction to derive a parsimonious model. The resulting coefficients informed construction of the nomogram.

Model performance was assessed by: (i) discrimination, quantified as the Area Under the Receiver Operating Characteristic Curve (AUC) with bootstrap 95% Confidence Intervals (CIs, 2000 resamples); (ii) calibration, assessed by calibration intercept, calibration slope, and Brier score with graphical calibration plots; and (iii) clinical utility, evaluated by Decision Curve Analysis (DCA) across threshold probabilities of 0.10–0.70. To approximate prospective transportability, we performed temporal validation, training on the earlier 70% of accrual (by admission date) and validating on the later 30%, reporting AUC, Brier score, calibration intercept/slope, Hosmer–Lemeshow (HL) *p*-value, and DCA.

We prespecified a clinical threshold of 0.30 and reported sensitivity, specificity, Positive Predictive Value (PPV), Negative Predictive Value (NPV), and confusion matrices in both cohorts at this fixed threshold. Operating thresholds were further summarized on the validation cohort across *p* = 0.05–0.60 (*Δ* = 0.05), with sensitivity, specificity, PPV/NPV, F1 score, Youden index, and Positive/Negative Likelihood Ratios (LR+/LR−). Net benefit was assessed using DCA. Internal validation employed 1,000-bootstrap optimism correction.

For benchmarking, we constructed two reproducible baselines [ASIA-only; ASIA + Neurological Level of Injury (NLI)] using the same split and preprocessing; performance, calibration, DCA, and reclassification metrics [Net Reclassification Improvement (NRI), Integrated Discrimination Improvement (IDI)] are summarized in the Supplement. Calibration drift was examined in the validation set; when observed, we applied intercept–slope updating and reported pre/post metrics.

All analyses were conducted in R (version 4.2.0; R Foundation for Statistical Computing, Vienna, Austria) using standard packages (glmnet, pROC, rms, rmda, boot). A two-tailed *p* < 0.05 was considered statistically significant.

## Results

### Study population and flow

From January 1, 2018 to March 31, 2023, we identified 308 eligible ICU TCSCI patients (Figure 1) and performed an outcome-stratified 7:3 split into a training cohort (*n* = 215; tracheostomy 73/215, 34.0%) and a validation cohort (*n* = 93; tracheostomy 32/93, 34.4%). Detailed exclusions and the study flow are presented in Figure 1.

### Baseline characteristics (training cohort)

In the training cohort (*n* = 215), baseline demographic and clinical features are summarized in Table 1, and univariable comparisons between patients with vs. without tracheostomy are reported in Table 2. Exploratory analyses showed higher tracheostomy rates among patients with BMI ≥ 25 kg/m^2^, smoking history, cervical dislocation, ISS ≥ 16, high cervical level (C1–C4), thoracic injury, and ASIA grade A (all *p* < 0.05).

**Table 1:** Characteristics of patients in the training and validation cohorts.

Characteristic	Training group (*n* = 215)	Validation group (*n* = 93)
Sex		
Male	184 (85.6)	84 (90.3)
Female	31 (14.4)	9 (9.7)
Age (yr)		
≥60	98 (45.6)	42 (45.2)
<60	117 (54.4)	51 (54.8)
BMI		
<25	165 (76.7)	67 (72.0)
≥25	50 (23.3)	26 (28.0)
Smoking history		
Yes	123 (57.2)	45 (48.4)
No	92 (42.8)	48 (51.6)
Dislocation		
Yes	171 (79.5)	74 (79.6)
No	44 (20.5)	19 (20.4)
ISS		
<16	109 (50.7)	44 (47.3)
≥16	106 (49.3)	49 (52.7)
Diabetes mellitus		
Yes	16 (7.4)	7 (7.5)
No	199 (92.6)	86 (92.5)
Hypertension		
Yes	51 (23.7)	28 (30.1)
No	164 (76.3)	65 (69.9)
ASIA impairment scale		
A	53 (24.7)	22 (23.7)
B-D	162 (75.3)	71 (76.3)
Neurological level of injury		
C1-4	105 (48.8)	28 (30.1)
C5-8	110 (51.2)	65 (69.9)
Preexisting lung disease		
Yes	7 (3.3)	1 (1.1)
No	208 (96.7)	92 (98.9)
Brain injury		
Yes	77 (35.8)	17 (18.3)
No	138 (64.2)	76 (81.7)
Thoracic injury		
Yes	97 (45.1)	36 (38.7)
No	118 (54.9)	57 (61.3)
Operation		
Yes	171 (79.5)	77 (82.8)
No	44 (20.5)	16 (17.2)
Mannitol		
Yes	181 (84.2)	79 (84.9)
No	34 (15.8)	14 (15.1)
Corticosteroids		
Yes	186 (86.5)	77 (82.8)
No	29 (13.5)	16 (17.2)
Tracheostomy		
Yes	73 (34.0)	32 (34.4)
No	142 (66.0)	61 (65.6)

**Table 2:** Comparison between patients with and without tracheostomy in the training cohort.

Variable	Tracheostomy (*n* = 73)	Without tracheostomy (*n* = 142)	*p*-value
Sex			0.546
Male	61 (83.6)	123 (86.6)	
Female	12 (16.4)	19 (13.4)	
Age (yr)			0.344
≥60	30 (41.1)	68 (47.9)	
<60	43 (58.9)	74 (42.1)	
BMI			0.006
<25	48 (65.8)	117 (82.4)	
≥25	25 (34.2)	25 (17.6)	
Smoking history			<0.001
Yes	54 (74.0)	69 (48.6)	
No	19 (26.0)	73 (51.4)	
Dislocation			0.005
Yes	66 (90.4)	105 (73.9)	
No	7 (9.6)	37 (26.1)	
ISS			<0.001
<16	23 (31.5)	86 (60.6)	
≥16	50 (68.5)	56 (39.4)	
Diabetes mellitus			0.432
Yes	4 (5.5)	12 (8.5)	
No	69 (94.5)	130 (91.5)	
Hypertension			0.144
Yes	13 (17.8)	38 (26.8)	
No	60 (82.2)	104 (73.2)	
ASIA impairment scale			<0.001
A	41 (56.2)	12 (8.5)	
B–D	32 (43.8)	130 (91.5)	
Neurological level of injury			0.002
C1-4	25 (34.2)	80 (56.3)	
C5-8	48 (65.8)	62 (43.7)	
Preexisting lung disease			1.000
Yes	2 (2.7)	5 (3.5)	
No	71 (97.3)	137 (96.5)	
Brain injury			0.391
Yes	29 (39.7)	48 (33.8)	
No	44 (60.3)	94 (66.2)	
Thoracic injury			0.041
Yes	40 (54.8)	57 (40.1)	
No	33 (45.2)	85 (59.9)	
Operation			0.013
Yes	65 (89.0)	106 (74.6)	
No	8 (11.0)	36 (25.4)	
Mannitol			0.315
Yes	66 (87.7)	117 (82.4)	
No	7 (12.3)	25 (17.6)	
Corticosteroids			0.364
Yes	61 (83.6)	125 (88.0)	
No	12 (16.4)	17 (12.0)	

Univariable comparisons within the training cohort. *p*-values from *χ*^2^ or Fisher’s exact tests. ASIA coded A = 0, B–D = 1.

### Predictor selection and multivariable model

**Figure 2:**
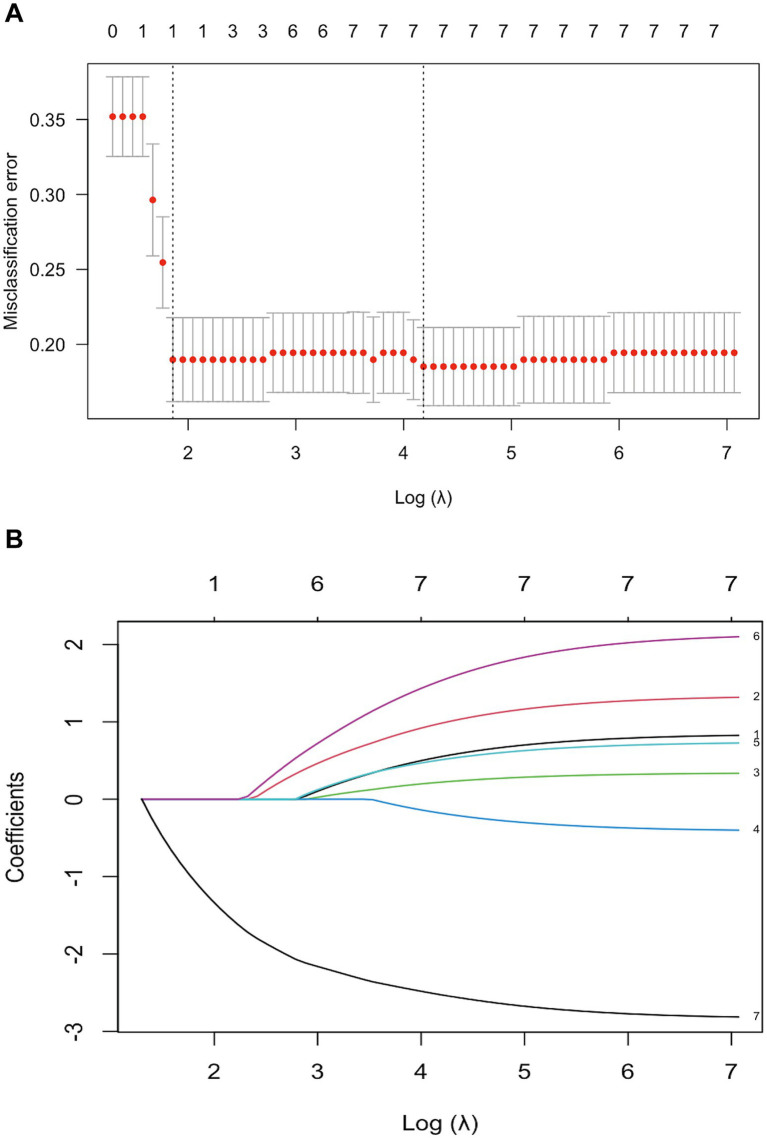
**(A)** LASSO regression model for predictor selection. The optimal parameter (lambda) was determined by plotting the binomial deviance curve against log(lambda), with dotted vertical lines based on the 1-standard error criterion. **(B)** Coefficient profile plot against the log(lambda) sequence. Variables: 1, Smoking history; 2, BMI; 3, ISS; 4, Neurological level of injury; 5, Thoracic injury; 6, Dislocation; 7, ASIA. Seven variables with nonzero coefficients were selected at the optimal lambda.

**Figure 3:**
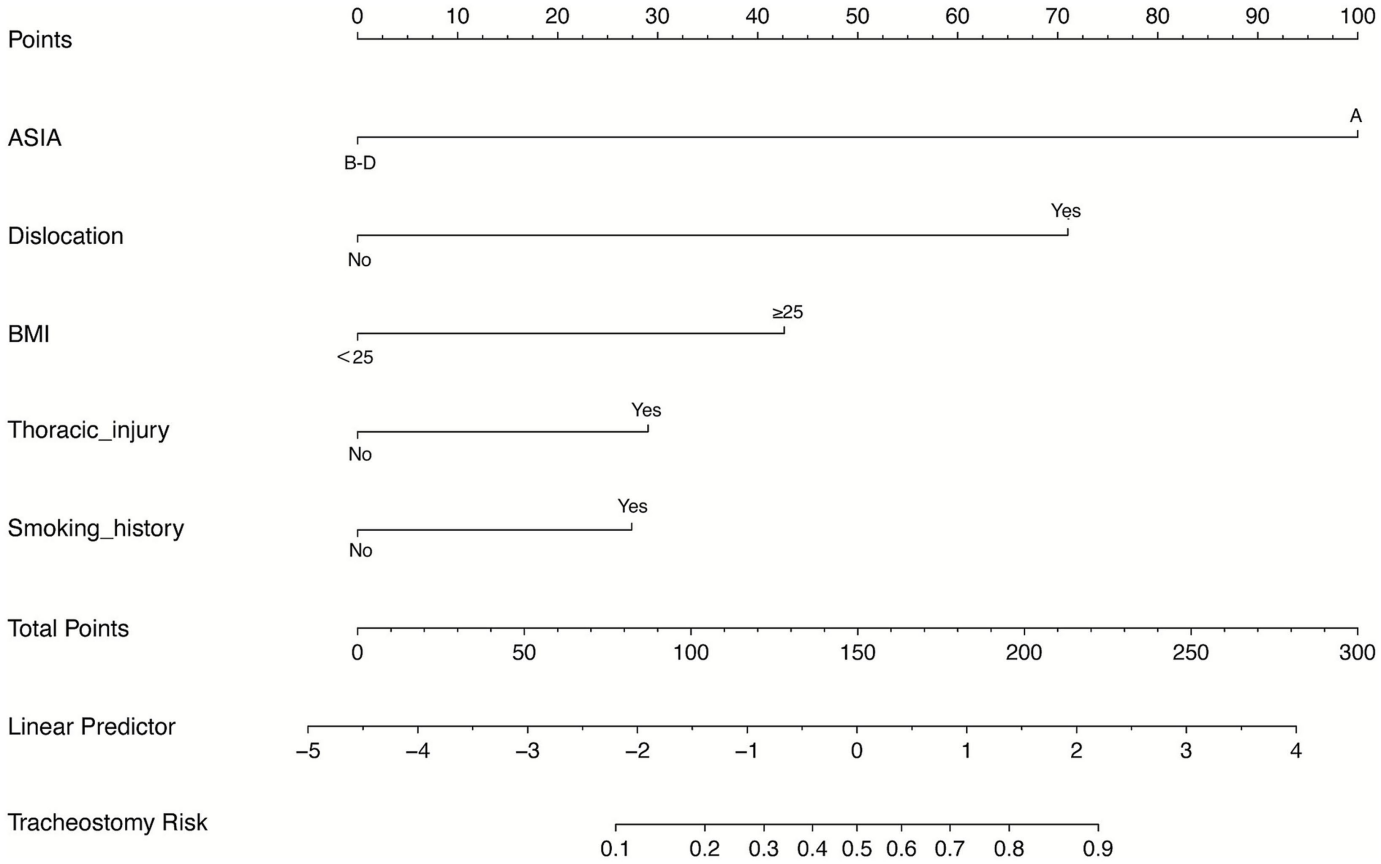
Nomogram model for predicting tracheostomy.

LASSO identified seven non-zero admission predictors—smoking history, BMI, ISS, NLI, thoracic injury, cervical dislocation, and ASIA grade (Figure 2). These were entered into a multivariable logistic regression, from which five independent predictors remained: smoking history (OR 2.40, 95% CI 1.09–5.28), thoracic injury (2.48, 1.15–5.31), BMI ≥ 25 kg/m^2^ (3.57, 1.55–8.20), cervical dislocation (8.75, 2.40–31.92), and ASIA (B–D vs. A: OR 0.042, 95% CI 0.016–0.111). For clinical interpretability, this corresponds to ASIA A vs. B–D: OR ≈ 23.81 (95% CI ≈ 9.01–62.50). A nomogram based on these predictors was constructed (Figure 3).

### Model performance (training cohort)

**Figure 4:**
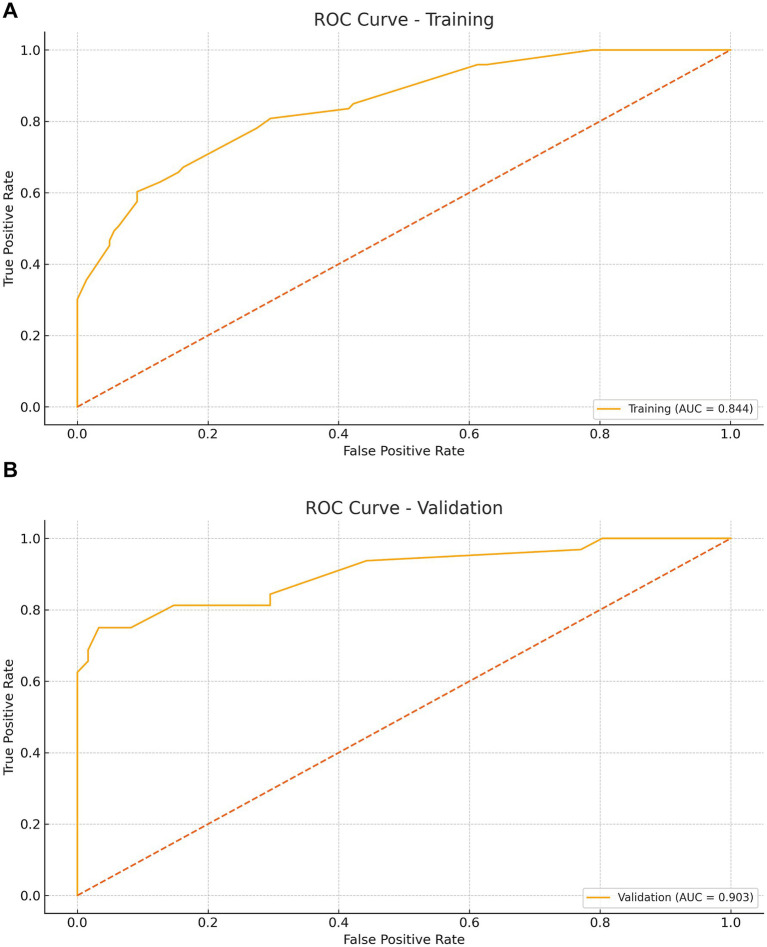
Receiver operating characteristic (ROC) curves for the nomogram model. Panel **(A)** shows the ROC curve for the training cohort (AUC = 0.844, 95 % CI 0.788–0.896), and Panel **(B)** shows the ROC curve for the validation cohort (AUC = 0.903, 95 % CI 0.823–0.966). Shaded bands indicate bootstrap 95 % confidence intervals.

**Figure 5:**
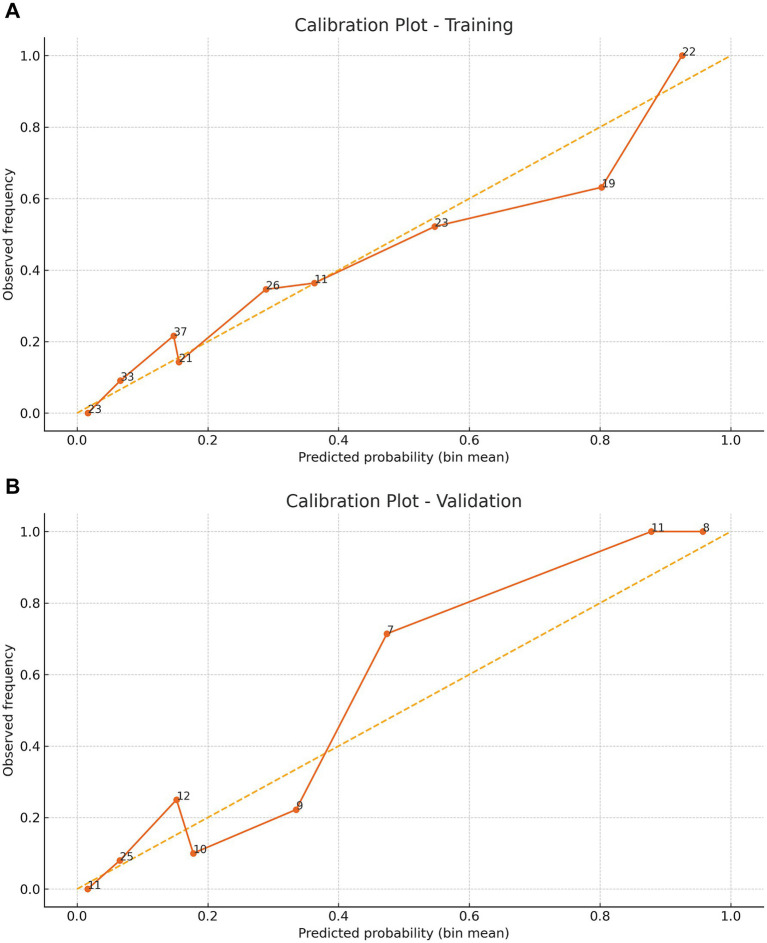
Calibration curves demonstrating the predictive performance stability of the nomogram. Panel **(A)** shows the apparent and bootstrap‑corrected calibration curves (1,000 resamples) for the training cohort, and Panel **(B)** shows the corresponding curves for the validation cohort. The inset lists the calibration intercept, slope and Brier score.

**Figure 6:**
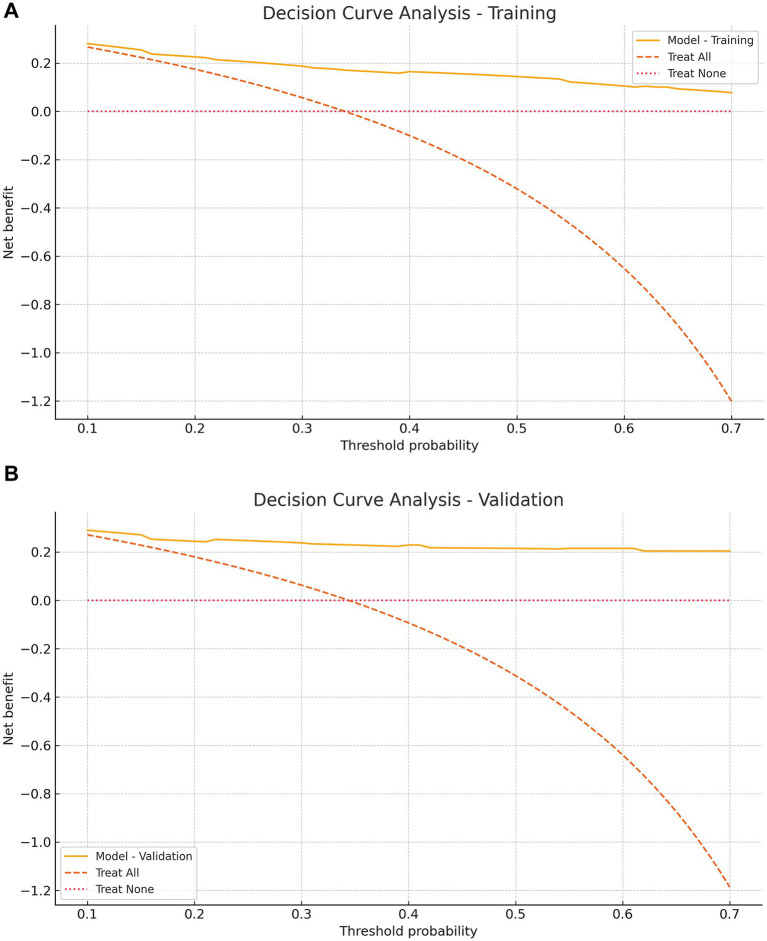
Decision curve analysis (DCA) for the training group **(A)** and validation group **(B)**. Net benefit of the nomogram versus “treat all” and “treat none” across threshold probabilities 0.10–0.70; the model confers higher net benefit in the clinically relevant range (~0.2–0.6).

The model showed strong discrimination (AUC 0.844, 95% CI 0.788–0.896; Figure 4A). Calibration was good (intercept 0.007, slope 0.901, Brier 0.144; Figure 5A). Using a prespecified clinical threshold of 0.30, performance was: True positives (TP) 57, false positives (FP) 39, true negatives (TN) 103, and false negatives (FN) 16, yielding Sensitivity 0.781, Specificity 0.725, PPV 0.594, and NPV 0.866. DCA showed greater net benefit than “treat all” or “treat none” across threshold 0.10–0.70 (Figure 6A).

### Temporal (time-split) validation

In the prespecified late validation window, discrimination, calibration, and net benefit were comparable to those in the main split-sample analysis (Supplementary Figures S1–S3; Supplementary Table S1), supporting stable temporal transportability. Multivariable logistic regression results (Table 3) showed that smoking history, thoracic injury, BMI ≥ 25 kg/m², cervical dislocation and ASIA grade B–D were independent predictors of tracheostomy risk.

**Table 3:** Multivariable logistic regression analysis.

Intercept and variable	*β*	Wald	*p*-value	OR	95% CI
Intercept	−1.596	4.296	0.038	0.203	0.0421–0.883
Smoking history (yes)	0.875	4.746	0.029	2.400	1.092–5.275
Thoracic injury (yes)	0.906	5.424	0.019	2.475	1.154–5.306
BMI ≥ 25 kg/m^2^	1.272	8.968	0.002	3.568	1.552–8.202
Dislocation (yes)	2.169	10.802	0.001	8.753	2.400–31.916
ASIA (B–D vs. A)	−3.166	40.930	<0.001	0.042	0.016–0.111

Multivariable logistic regression after LASSO preselection. Coding: ASIA (A = 0, B–D = 1). ORs shown for B–D vs A; in text we report ASIA A vs B–D by reciprocal for clinical interpretability.

### Validation cohort

In the validation cohort (*n* = 93), 32 patients (34.4%) underwent tracheostomy. Discrimination remained high (AUC 0.903, 95% CI 0.823–0.966; Figure 4B). Calibration was acceptable to good (intercept 0.577, slope 1.275, Brier 0.098; Figure 5B). At the 0.30 threshold, performance was: TP 26, FP 9, TN 52, and FN 6, yielding Sensitivity 0.812, Specificity 0.852, PPV 0.743, and NPV 0.897. DCA again demonstrated greater net benefit than “treat all/none” across thresholds 0.10–0.70 (Figure 6B).

Threshold-wise performance and decision-curve net benefit are provided for clinical selection of operating points (Supplementary Table S2; Supplementary Figure S4), enabling high-sensitivity, resource-constrained, or balanced operating strategies.

Head-to-head benchmarking showed that the nomogram outperformed ASIA-only and ASIA + NLI baselines in AUC, calibration, and net benefit across clinically relevant thresholds; category-free NRI/IDI likewise favored the nomogram (Supplementary Table S3; Supplementary Figures S5–S7).

After intercept–slope recalibration on the validation set, absolute risk alignment improved while discrimination remained unchanged (Supplementary Table S4; Supplementary Figure S8).

## Discussion

We developed and internally validated a nomogram to estimate the probability of tracheostomy in patients with TCSCI, using variables available upon ICU admission. Five independent predictors were identified: smoking history, thoracic injury, BMI ≥ 25 kg/m^2^, cervical dislocation, and ASIA grade. The model demonstrated robust performance, with strong discrimination (AUC 0.844, 95% CI 0.788–0.896 in training; 0.903, 95% CI 0.823–0.966 in validation), favorable calibration (training intercept 0.007, slope 0.901; validation intercept 0.577, slope 1.275), and low prediction error (Brier scores: 0.144 and 0.098, respectively). Across a threshold probability range of 0.10–0.70, the nomogram consistently yielded higher net benefit than “treat all” or “treat none” strategies. At a clinically prespecified cutoff of 0.30, model performance remained well balanced (training: sensitivity 0.781, specificity 0.725; validation: sensitivity 0.812, specificity 0.852).

Among the predictors identified, ASIA grade A emerged as the most powerful independent predictor of tracheostomy. This aligns with prior studies demonstrating that complete neurological impairment is associated with a markedly elevated tracheostomy risk (OR ≈14.2), often with high specificity and a low false-positive rate (21, 22). Similarly, CART-based models have consistently emphasized the predominant role of complete cord injury in determining tracheostomy need (23). Our findings reaffirm these results, with ASIA grade A scoring highest in the nomogram, underscoring its central role in airway management planning (24).

Another notable finding is the identification of cervical facet dislocation as a significant risk factor. Patients with facet dislocations typically present with more severe neurological deficits and poorer motor recovery potential (25). Approximately 75% of such patients are classified as ASIA A or B, reflecting both injury severity and respiratory vulnerability (26). Our results echo previous nomograms incorporating ASIA, neurological level, dislocation, smoking, and thoracic injury, highlighting facet dislocation’s key role in cord compression and tracheostomy risk (24). These findings are consistent with Mu et al. (8), who also identified facet dislocation as an independent predictor of tracheostomy in TCSCI.

The association between age and tracheostomy need in TCSCI remains controversial. Some studies suggest that older age (e.g., >50 or >69 years) predicts lower ventilator weaning success and increased tracheostomy risk (27, 28), whereas others found no significant link between age and airway complications (29, 30). In our cohort, age did not emerge as an independent predictor, possibly due to confounding by ASIA grade or comorbidities. The adequate sample size and adjustment for neurological status may have attenuated age effects. This discrepancy underscores the need for larger, multicenter studies to clarify age-related airway outcomes following TCSCI.

In addition to ASIA grade and cervical dislocation, smoking history, thoracic injury, and BMI were also identified as independent predictors of tracheostomy. These findings align with previous studies demonstrating that nomogram models incorporating ASIA grade, neurological level of injury, smoking history, dislocation, and thoracic injury can reliably predict tracheostomy risk in TCSCI patients (24). A 10-year study further supported this by identifying smoking history as an independent risk factor for tracheostomy after TCSCI (21). Smoking is known to increase susceptibility to pulmonary infections and chronic lung diseases induced by cigarette smoke (31). Similarly, Nakashima et al. (32) also reported smoking history as a significant risk factor for tracheostomy. The inclusion of BMI as an independent predictor introduces a novel and clinically relevant dimension. Elevated BMI may be associated with reduced pulmonary reserve and impaired airway clearance, potentially predisposing patients to prolonged ventilatory dependence.

The validated nomogram provides a practical and accessible tool for early risk stratification of tracheostomy in TCSCI patients. Its high sensitivity enables timely identification of high-risk individuals, facilitating earlier respiratory specialist consultation and more efficient resource allocation in trauma care settings. Early detection of at-risk patients may reduce complications related to delayed tracheostomy, such as prolonged mechanical ventilation and ventilator-associated pneumonia (6, 9). The use of routinely available clinical variables enhances the nomogram’s feasibility for real-world implementation across diverse healthcare systems. Integration into electronic health record systems could enable automated risk calculation, streamline clinical workflows, and support precision medicine approaches in TCSCI management. Moreover, the model can serve as a communication aid, providing individualized risk estimates to guide patient and family counseling and support shared decision-making.

## Strengths and limitations

This study has several strengths, including transparent modeling procedures, prespecified clinical thresholds, and comprehensive reporting—encompassing bootstrap confidence intervals, calibration intercepts/slopes, Brier scores, and decision-curve analyses—which collectively enhance interpretability and reproducibility. The inclusion of ASIA A, facet dislocation, concomitant thoracic injuries, BMI, and smoking status in the final model is clinically sound and reflects a clear understanding of factors that influence airway management in TCSCI. However, the model did not incorporate dynamic respiratory parameters or diaphragmatic ultrasound—physiological measures that may capture the evolution of respiratory function and add incremental predictive value. The single-center, retrospective design raises the possibility of selection bias, and the modest validation cohort may limit generalizability. Mild miscalibration also suggests that further model updating may be required. Although temporal validation indicated stable performance over time, external validation across multiple centers remains essential. Additional variables such as sedation strategies and early rehabilitation were not available and may contribute to residual confounding. Future work should prioritize prospective, multicenter validation and the implementation of risk-guided tracheostomy pathways, incorporating dynamic physiological data and periodic recalibration to maximize clinical utility.

## Conclusion

In summary, our findings support the development of a clinically applicable, bedside-friendly nomogram for early prediction of tracheostomy in patients with TCSCI. The model demonstrates strong discriminative power, reliable calibration, and potential for integration into clinical decision support systems. Although further prospective and multicenter validations are warranted, this nomogram represents a promising tool to facilitate individualized airway management strategies in acute spinal trauma care.

## Data Availability

The original contributions presented in the study are included in the article/Supplementary material, further inquiries can be directed to the corresponding authors.
