# Supplementary material for: Development and validation of a nomogram for predicting tracheostomy risk in traumatic cervical spinal cord injury
Source: Front Neurol. 2026 Jan 15;16:1684974. doi: 10.3389/fneur.2025.1684974 (PMC12852336; doi:10.3389/fneur.2025.1684974)
Supplement: Supplementary file 1 [file Data_Sheet_1.pdf]

Ethical review approval from the Human Research Ethics Committee of the Second Affiliated Hospital of Zhejiang University School of Medicine

Project acceptance number: Yan 2023-0287 Event acceptance number: I2023352 Approval number: (2023) Lun Shen Yan No. (0373)

project name	Investigation of traumatic cervical spinal cord injury in Hangzhou		
Type of application	Registration studies: ☐ Drug clinical trials and device clinical trials ☐ Clinical trials of special medical foods<br>Non-registration studies: post-marketing product studies in clinical research areas		
Classification of drugs/medical devices		Test phases	
The Chinese side	The Second Affiliated Hospital of Zhejiang University School of Medicine		
CRO			
Responsible departments	Critical care medicine	Principal Investigator PI	Zhang Gensheng / Chen Weiting
District Fuzikou participated	Team leader unit: /	Team leaders unit PI	/
audit-review file			
order number	Name of material	version number	Language/Version date
1	Human research request form of Zhejiang University Second Hospital		the Chinese language /
2.	Clinical study protocol	the front page	Chinese/2023
3	Medical report form		the Chinese language /
4	GCP certificate of training		the Chinese language
5	Resume of the main researcher and brief introduction of participants		the Chinese language
6	Researcher position description		the Chinese language
7	Letter of commitment from the person in charge of the clinical research project		the Chinese language !
8	Informed consent waiver form		the Chinese language /
Form of review	This review: ☐ The meeting review is a rapid review		
Review date	This review date: 2023-04-12	congress venue	/
juror	Yin Xinzhen Chen Zexin		

Conclusions of the review	1. After review by the Human Ethics Committee, the result is: agree. Opinions and suggestions:<br>2. During the course of this study, the research will be subject to follow-up review by this Ethics Review Committee at the frequency of approval of the study<br>Start: ☐ 6 months, 12 months, etc

Signature of Director/Deputy Director: 吴志英

Period: 2023/04/12

st[illegible] n

(affix [illegible] seal)

Personnel of the Second Affiliated Hospital of Zhejiang University School of Medicine

Research Notes:

1. In the study, please abide by the principles of GCP and the Helsinki Declaration,

2. The research shall be carried out in strict accordance with the approved protocol. During the research, any modification of clinical research protocol, informed consent and other materials, as well as the change of the principal investigator, shall be submitted for modification application, which can be continued only after approval by the Ethics Committee;

3. Please submit the application for follow-up review one month in advance, and this ethics committee will make a new decision based on the results of follow-up review.

4. Serious adverse events shall be reported in writing as required, and the Ethics Committee shall make a review decision based on the serious adverse events report. 5. Written reports shall be provided for any plan violation/deviation, suspension or termination.

6. Provide a summary report at the end of the study.

7. Report important decisions of the centers ethics in writing in a timely manner;

List of human research ethics committee members of the Second Affiliated Hospital of Zhejiang University School of Medicine					
Ethics Committee functions	surname and personal name	sex	specialty	professional ranks and titles	work unit
chairman	Wu Zhiying	woman	neurology	professor	Zhejiang University Second Hospital
vice chairman	Hu Xinyang	woman	Cardiovascular and internal medicine	botanic physician	Zhejiang University Second Hospital
committee member	Lin Zheng	man	psychiatry	botanic physician	Zhejiang University Second Hospital
committee member	Dai Haibin	man	pharmaceutical preparation section	pharmacist	Zhejiang University Second Hospital
committee member	Zhu Junming	man	neurosurgery	botanic physician	Zhejiang University Second Hospital
committee member	Jin Xiuming	man	ophthalmology	botanic physician	Zhejiang University Second Hospital
committee member	Yao Meiqi	woman	nursing	co-chief superintendent nurse	Zhejiang University Second Hospital
committee member	Satellite imagery	man	Medical ethics	professor	Zhejiang University Second Hospital
committee member	Xu Jiangling	man	jurisprudence	lawyer	Guohao Law Group (Hangzhou Office So)
committee member	Shi Chongyi	man	Cultural and institutional management	Senior political engineer	Zhejiang Opera House
committee member	Yu Hong	man	clinical research	professor	Zhejiang University Second Hospital
committee member	Xu Rongzhen	man	Blood Department	professor	Zhejiang University Second Hospital
committee	Chen Zexin	man	Epidemiology and biostatistics	actuary	Zhejiang University

committee member					Second Hospital
committee member	Xue Jing	woman	rheumatology department	botanic physician	Zhejiang University Second Hospital
committee member	Li Jiangtao	man	general surgery department	botanic physician	Zhejiang University Second Hospital
committee member	Dong Ying	woman	medical oncology	botanic physician	Zhejiang University Second Hospital
committee member	Yin Xinzhen	woman	neurology	associate chief physician	Zhejiang University Second Hospital
committee member	Cheng Haiying	woman	paediatrics	associate chief physician	Zhejiang University Second Hospital
committee member	Tan Yanbin	man	orthopaedics and tranmstology	associate chief physician	Zhejiang University Second Hospital
committee member	Lanfing	woman	respiratory medicine department	associate chief physician	Zhejiang University Second Hospital
committee member	Shen Hong	woman	medical oncology	botanic physician	Zhejiang University Second Hospital

The list of committee members shall be subject to the hospital appointment document

The composition and operation mode of the Human Research Ethics Committee of the Second Affiliated Hospital of Zhejiang University School of Medicine strictly follow the provisions of GCP (including ICH-GCP) and relevant laws and regulations to carry out various operations

directive rules 。

Address: No.88 Jiefang Road, Hangzhou, Zhejiang Province 310009 Tel: 0571-87783759 Fax: 0571-87783969

The Second Affiliated Hospital of Zhejiang University School of Medicine

Human Research Ethics Committee
