# Supplementary material for: Development and validation of a nomogram for predicting tracheostomy risk in traumatic cervical spinal cord injury
Source: Front Neurol. 2026 Jan 15;16:1684974. doi: 10.3389/fneur.2025.1684974 (PMC12852336; doi:10.3389/fneur.2025.1684974)
Supplement: Supplementary file 2 [file Data_Sheet_2.pdf]

# 浙江大学医学院附属第二医院人体研究伦理委员会伦理审查批件

项目受理号：研2023-0287　事件受理号：I2023352　　　　批件号：（2023）伦审研第（0373）号

| | | | |
|---|---|---|---|
| 项目名称 | 杭州市创伤性颈脊髓损伤的调查研究 | | |
| 申请类型 | 注册研究：☐ 药物临床试验 ☐ 器械临床试验 ☐ 特医食品临床试验<br>非注册研究：☒ 临床科研 ☐ 上市后产品研究 | | |
| 药/械分类 | / | 试验分期 | / |
| 申办方 | 浙江大学医学院附属第二医院 | | |
| CRO | / | | |
| 承担科室 | 重症医学科 | 主要研究者PI | 张根生/陈卫挺 |
| ☒ 负责 ☐ 参加 | 组长单位：/ | 组长单位PI | / |
| 审查文件 | | | |
| 序号 | 材料名称 | 版本号 | 语言/版本日期 |
| 1 | 浙大二院人体研究申请表 | | 中文 / |
| 2 | 临床研究方案 | 第一版 | 中文 /2023 |
| 3 | 病历报告表 | | 中文 / |
| 4 | GCP培训证书 | | 中文 |
| 5 | 主要研究者简历及参加人员简介 | | 中文 |
| 6 | 研究者岗位职责 | | 中文 |
| 7 | 临床研究项目负责人承诺书 | | 中文 / |
| 8 | 免除知情同意申请表 | | 中文 / |
| 审查形式 | 本次审查：☐ 会议审查 ☒ 快速审查 | | |
| 审查日期 | 本次审查日期：2023-04-12 | 会议地点 | / |
| 审查委员 | 殷鑫浈　陈泽鑫 | | |
| 审查结论 | 1. 经本人体伦理审查委员会审查，审查结果：同意。意见和建议：<br>2. 该研究进行过程中将接受本伦理审查委员会的跟踪审查，跟踪审查频率为研究批准之日起：☐ 6个月 ☒ 12个月 ☐ 其它＿＿＿＿＿； | | |

主任/副主任委员签名：吴志英　　　　日期：2023/04/12

浙江大学医学院附属第二医院人[illegible]（盖章）

浙江大学医学院附属第二医院人体研究伦理委员会 [illegible]

研究注意事项：

1. 在研究中请遵守GCP和《赫尔辛基宣言》的原则，
2. 严格遵循批准的方案开展研究，研究过程中对临床研究方案、知情同意书等材料的任何修改及主要研究者变更等，请提交修改申请，得到伦理委员会批准后方可继续实施，
3. 请提前1个月提交跟踪审查申请，本伦理委员会根据跟踪审查的结果作出新的决定。
4. 按要求书面上报严重不良事件，本伦理委员会将根据严重不良事件报告作出审查决定。
5. 方案违背/偏离、暂停/终止均应提供书面报告。
6. 研究结束提供总结报告。
7. 及时书面报告中心伦理的重要决定。

## 浙江大学医学院附属第二医院人体研究伦理委员名单

| 伦理委员会职务 | 姓名 | 性别 | 专业 | 职称 | 工作单位 |
|---|---|---|---|---|---|
| 主任委员 | 吴志英 | 女 | 神经内科 | 教授 | 浙大二院 |
| 副主任委员 | 胡新央 | 女 | 心血管内科学 | 主任医师 | 浙大二院 |
| 委员 | 林铮 | 男 | 精神科 | 主任医师 | 浙大二院 |
| 委员 | 戴海斌 | 男 | 药剂科 | 主任药师 | 浙大二院 |
| 委员 | 朱君明 | 男 | 神经外科学 | 主任医师 | 浙大二院 |
| 委员 | 晋秀明 | 男 | 眼科 | 主任医师 | 浙大二院 |
| 委员 | 姚梅琪 | 女 | 护理学 | 副主任护师 | 浙大二院 |
| 委员 | 施卫星 | 男 | 医学伦理 | 教授 | 浙大二院 |
| 委员 | 徐江陵 | 男 | 法律学 | 律师 | 国浩律师集团（杭州事务所） |
| 委员 | 史崇义 | 男 | 文化事业管理 | 高级政工师 | 浙江歌舞剧院 |
| 委员 | 余红 | 男 | 临床研究 | 教授 | 浙大二院 |
| 委员 | 徐荣臻 | 男 | 血液科 | 教授 | 浙大二院 |
| 委员 | 陈泽鑫 | 男 | 流行病与生物统计学 | 统计师 | 浙大二院 |
| 委员 | 薛静 | 女 | 风湿科 | 主任医师 | 浙大二院 |
| 委员 | 李江涛 | 男 | 普外科 | 主任医师 | 浙大二院 |
| 委员 | 董颖 | 女 | 肿瘤内科 | 主任医师 | 浙大二院 |
| 委员 | 殷鑫浈 | 女 | 神经内科 | 副主任医师 | 浙大二院 |
| 委员 | 程海英 | 女 | 儿科 | 副主任医师 | 浙大二院 |
| 委员 | 谭延斌 | 男 | 骨科学 | 副主任医师 | 浙大二院 |
| 委员 | 兰芬 | 女 | 呼吸内科 | 副主任医师 | 浙大二院 |
| 委员 | 沈虹 | 女 | 肿瘤内科 | 主任医师 | 浙大二院 |

委员名单根据医院聘任文件为准

浙江大学医学院附属第二医院人体研究伦理委员会的组成及操作方式严格遵循GCP（包括ICH-GCP）及相关法律和法规的规定，实施各项操作规程。

地　　址：浙江省杭州市解放路88号 310009 联系电话：0571－87783759 传真：0571－87783969

浙江大学医学院附属第二医院

人体研究伦理委员会
