# Supplementary figures and images for: Development and validation of a nomogram for predicting tracheostomy risk in traumatic cervical spinal cord injury
Source: Front Neurol. 2026 Jan 15;16:1684974. doi: 10.3389/fneur.2025.1684974 (PMC12852336; doi:10.3389/fneur.2025.1684974)

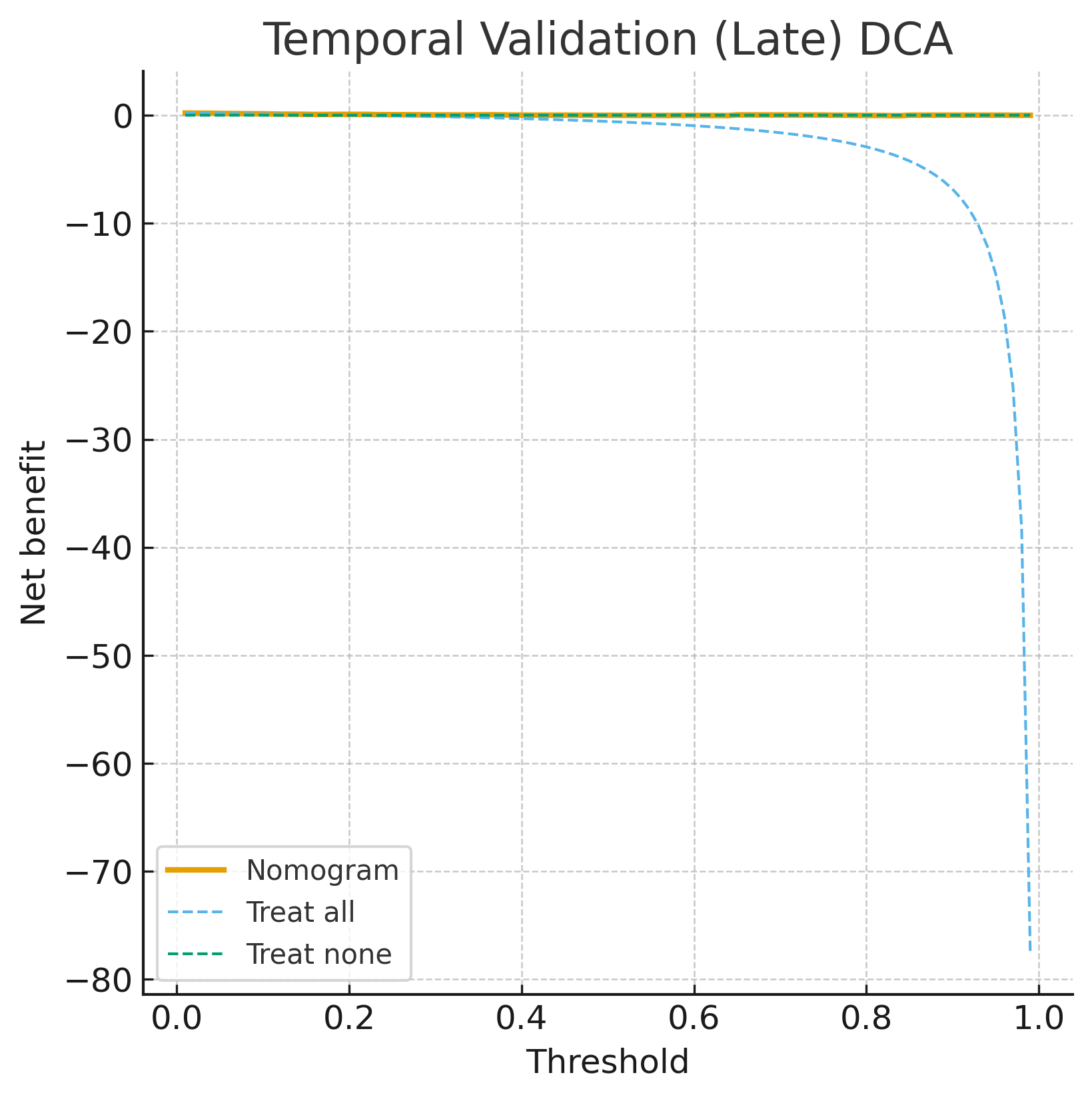

Supplement: Supplementary file 7 [file Image_1.tiff]

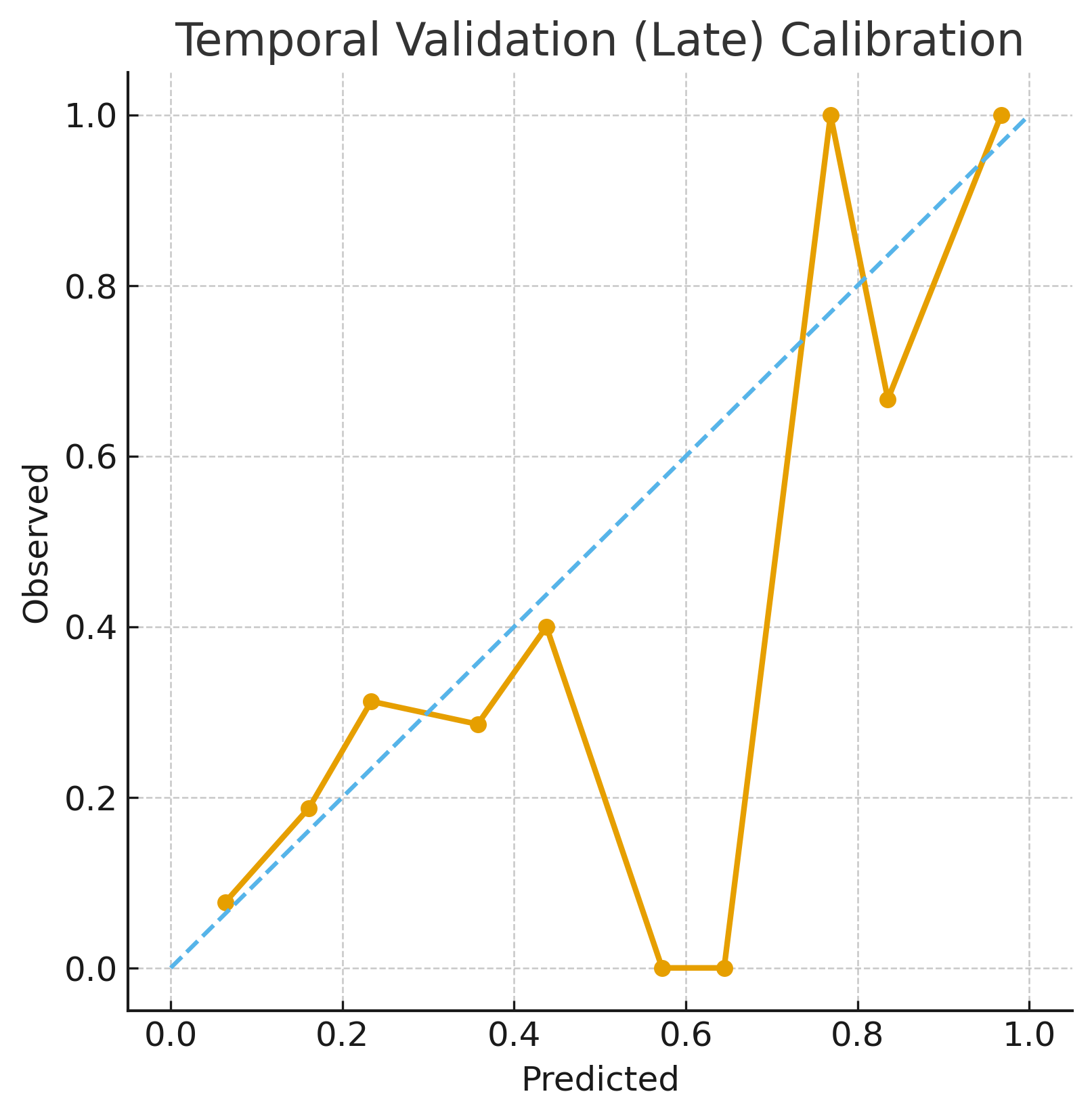

Supplement: Supplementary file 8 [file Image_2.tiff]

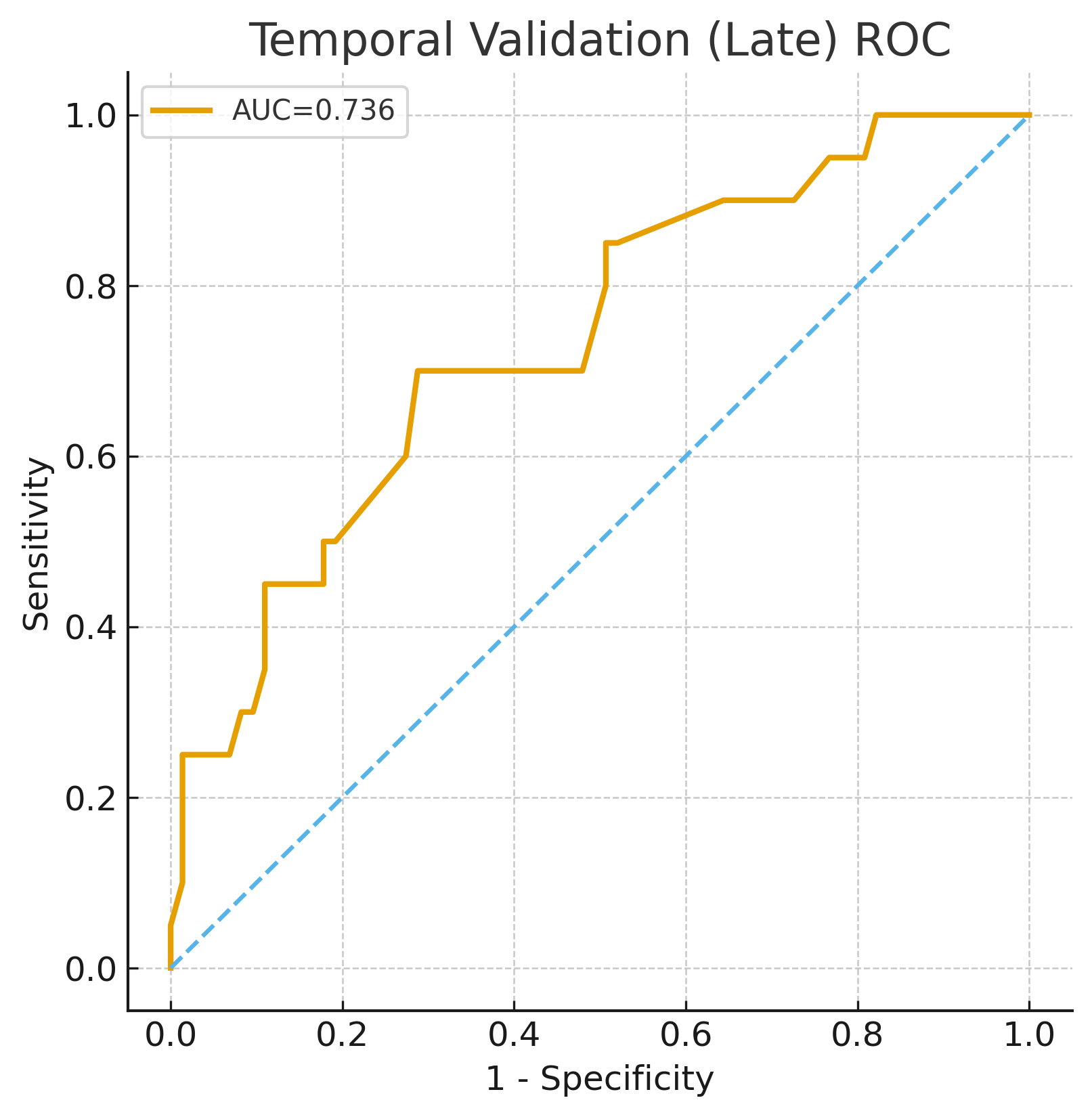

Supplement: Supplementary file 9 [file Image_3.tiff]

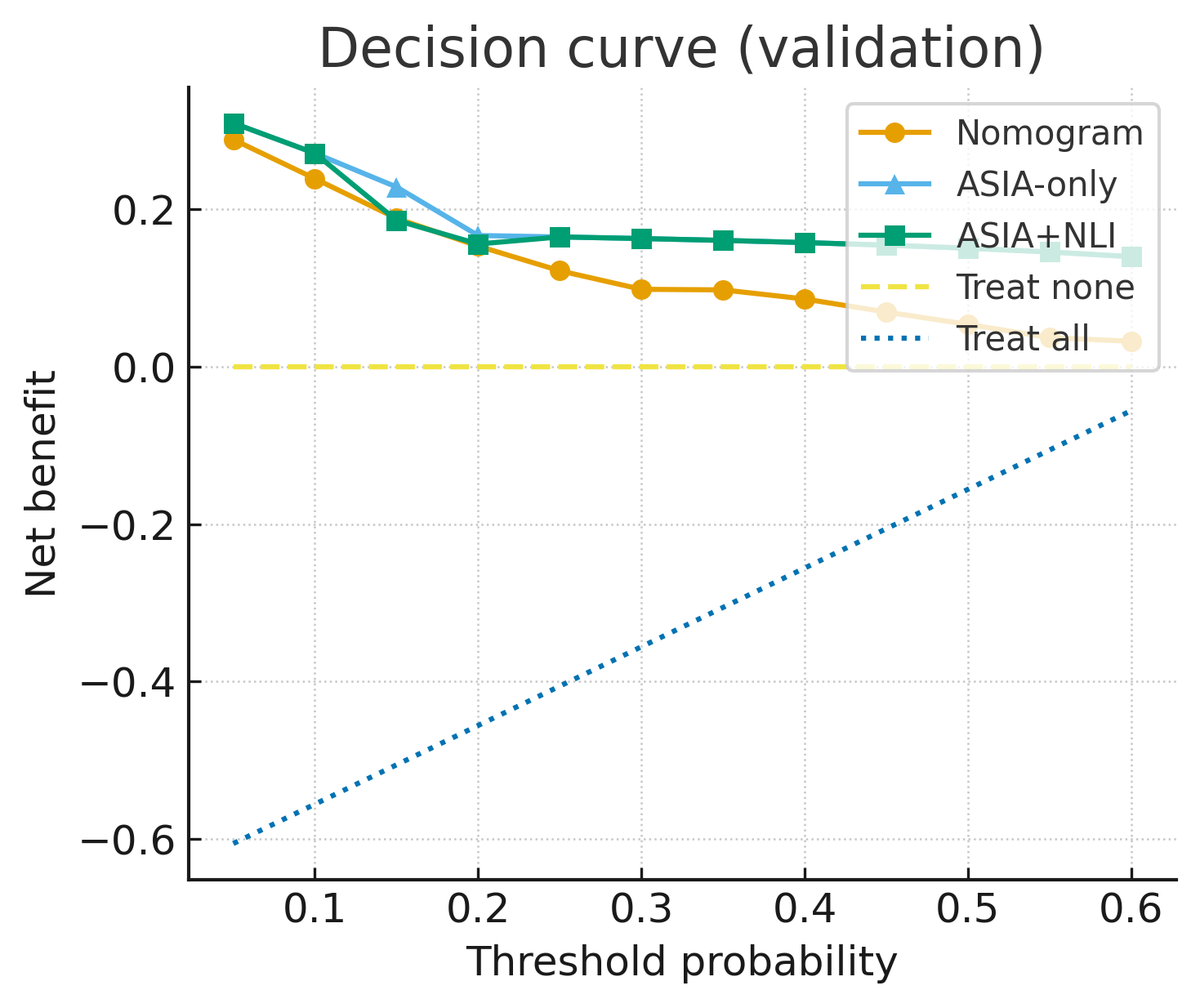

Supplement: Supplementary file 10 [file Image_4.tiff]

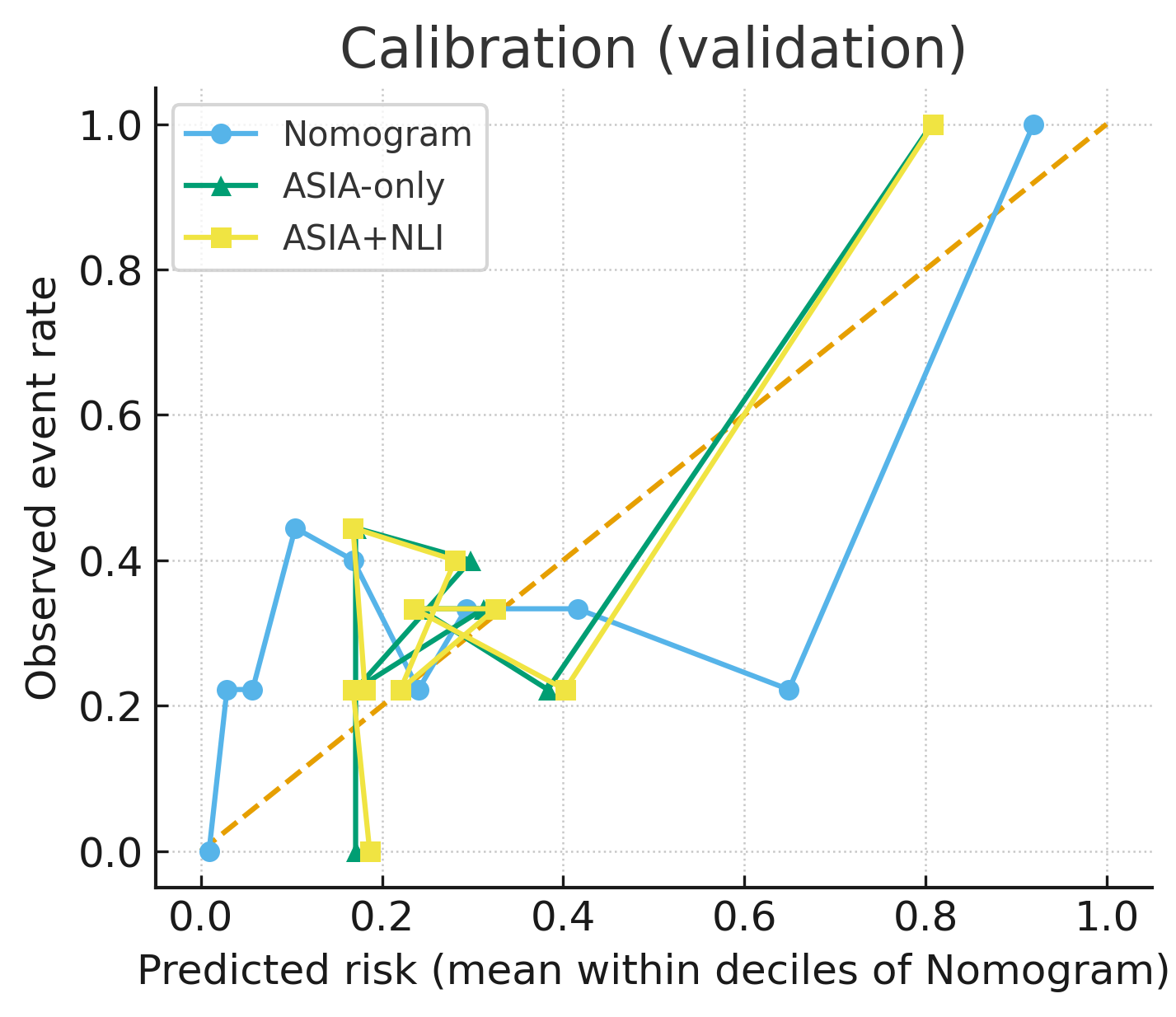

Supplement: Supplementary file 11 [file Image_5.tiff]

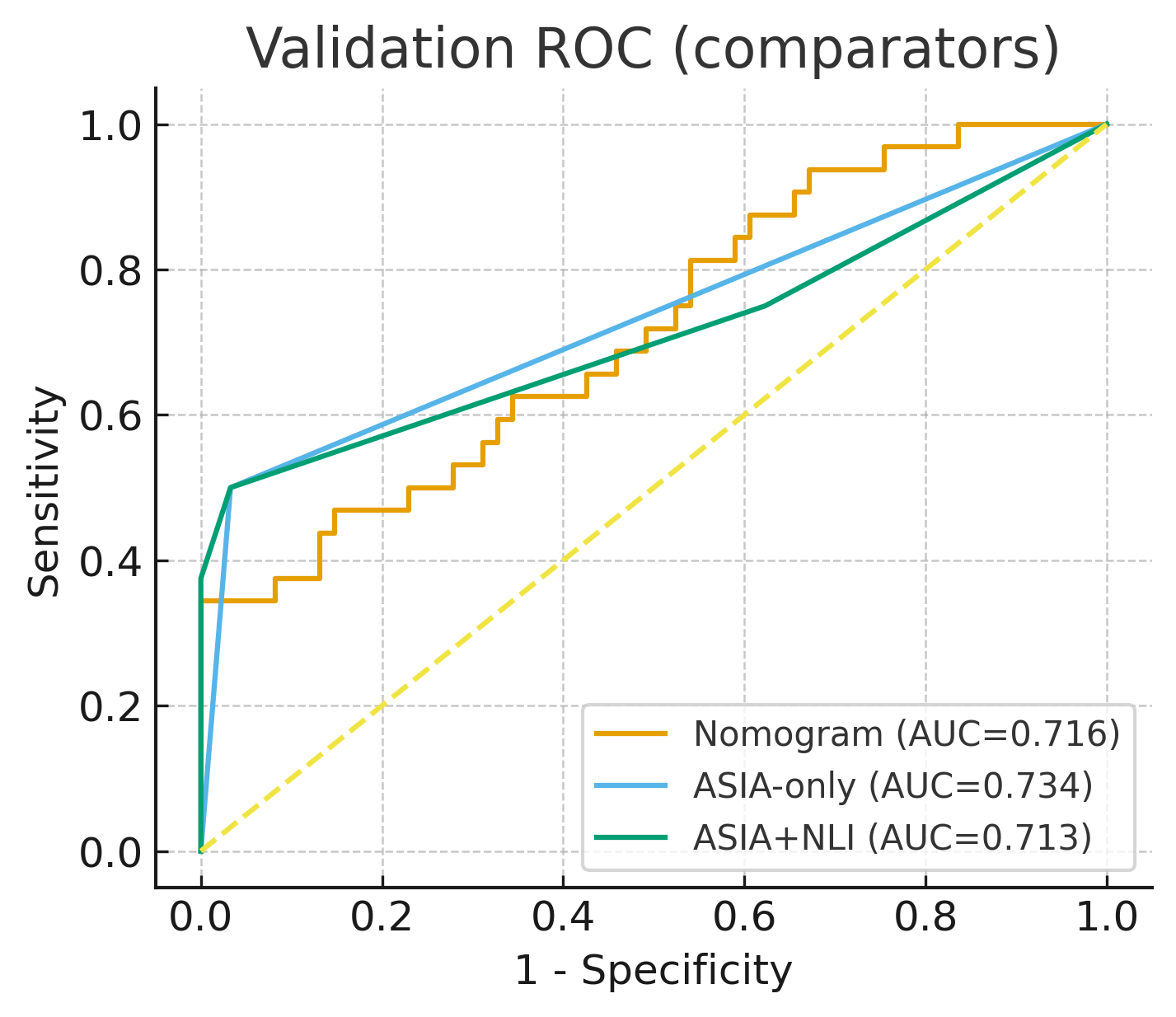

Supplement: Supplementary file 12 [file Image_6.tiff]

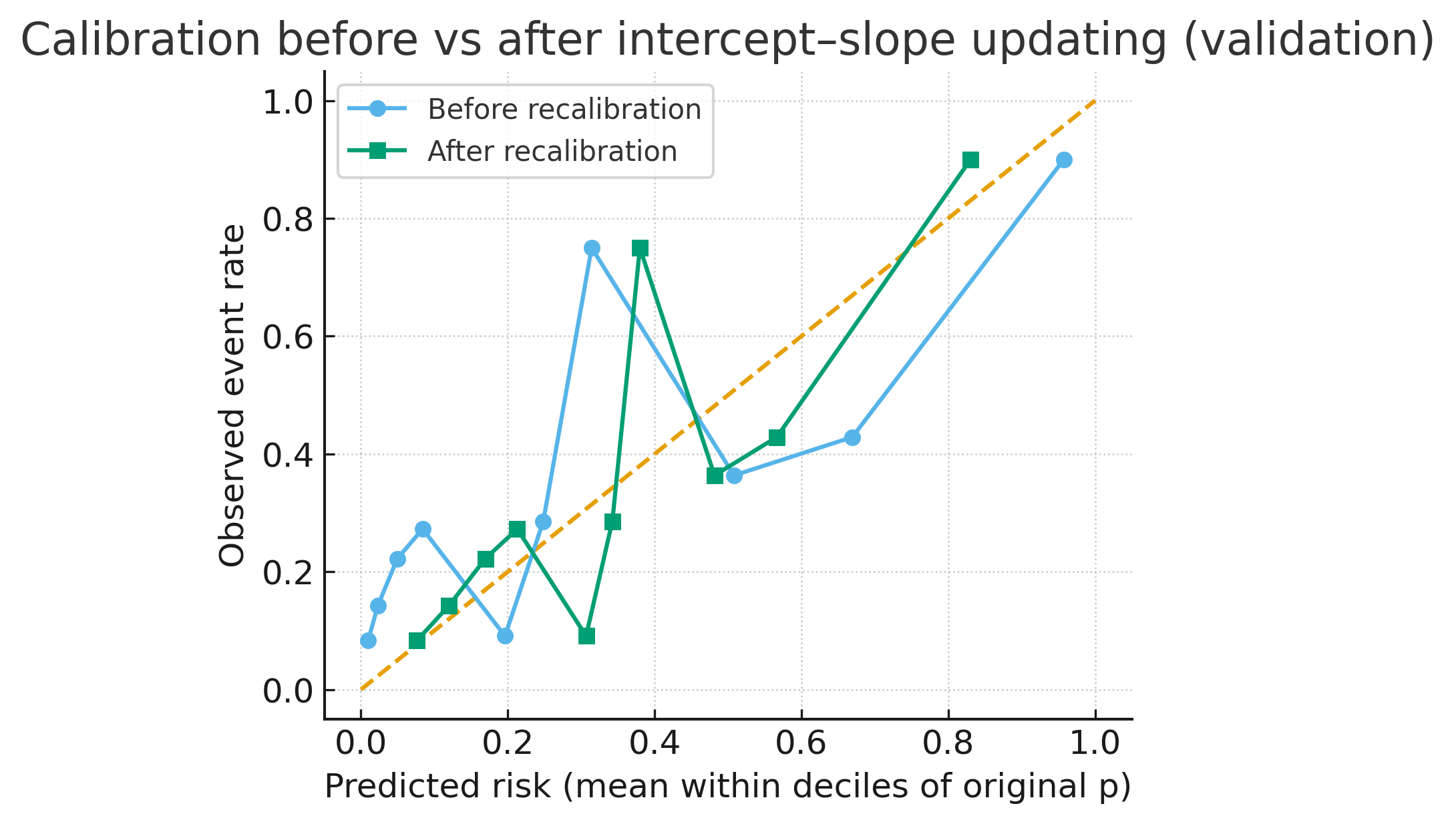

Supplement: Supplementary file 14 [file Image_8.tiff]
